# Chromosome-level assembly of the club-legged grasshopper (*Gomphocerus sibiricus*) genome

**DOI:** 10.1093/g3journal/jkaf231

**Published:** 2025-10-01

**Authors:** Octavio M Palacios-Gimenez, Mahendra Varma, Xinyi Cheng, Mai-Britt Mosbech, Alexander Suh, Holger Schielzeth

**Affiliations:** Population Ecology Group, Institute of Biodiversity, Ecology and Evolution, Friedrich Schiller University Jena, Dornburger Straße 159, Jena 07743, Thuringia, Germany; Department of Organismal Biology—Systematic Biology, Evolutionary Biology Centre, Uppsala University, Uppsala 75236, Uppland, Sweden; German Centre for Integrative Biodiversity Research (IDiv) Halle-Jena-Leipzig, Puschstraße 4, Leipzig 04103, Saxony, Germany; Population Ecology Group, Institute of Biodiversity, Ecology and Evolution, Friedrich Schiller University Jena, Dornburger Straße 159, Jena 07743, Thuringia, Germany; Max Planck Institute for Chemical Ecology, Hans-Knöll-Straße 8, Jena 07745, Thüringen, Germany; Population Ecology Group, Institute of Biodiversity, Ecology and Evolution, Friedrich Schiller University Jena, Dornburger Straße 159, Jena 07743, Thuringia, Germany; Max Planck Institute for Chemical Ecology, Hans-Knöll-Straße 8, Jena 07745, Thüringen, Germany; Department of Immunology, Genetics and Pathology, Uppsala Genome Center, Uppsala University, National Genomics Infrastructure Hosted by SciLifeLab, Box 518, Uppsala 75108, Uppland, Sweden; Department of Organismal Biology—Systematic Biology, Evolutionary Biology Centre, Uppsala University, Uppsala 75236, Uppland, Sweden; Centre for Molecular Biodiversity Research, Leibniz Institute for the Analysis of Biodiversity Change, Zoologisches Forschungsmuseum A. Koenig, Adenauerallee 160, Bonn 53113, North Rhine-Westphalia, Germany; Population Ecology Group, Institute of Biodiversity, Ecology and Evolution, Friedrich Schiller University Jena, Dornburger Straße 159, Jena 07743, Thuringia, Germany; German Centre for Integrative Biodiversity Research (IDiv) Halle-Jena-Leipzig, Puschstraße 4, Leipzig 04103, Saxony, Germany

**Keywords:** color polymorphism, chromosome-level genome assembly, Orthoptera, Acrididae, Gomphocerinae

## Abstract

Grasshoppers represent true outliers in genome sizes, both within insects and within animals in general. Their genomes are large and generally variable in sizes and feature a high abundance of repetitive DNA sequences. This has hampered the assembly of grasshopper genomes to the chromosome level. Here we present a chromosome-level reference genome for the club-legged grasshopper (*Gomphocerus sibiricus*, Acrididae: Gomphocerinae) using PacBio HiFi long-read and Hi-C sequencing technologies. In male haploid cells, the species has a chromosome set of n = 9 with an X0 sex-determination system, characterized by an absence of a Y chromosome. Our assembly spans 9.57 Gb in total, with 8.87 Gb organized into 9 chromosomes—8 autosomes and the X chromosome. The final assembly has a scaffold N50 value of 1.58 Gb, covers 96.7% single copy Insecta orthologs, and contains 42,665 predicted protein-coding genes and 43,385 mRNA transcripts. We compiled a curated, nonredundant, species-specific repeat library and used it to annotate repetitive DNA, covering 81.69% of the genome, mostly DNA transposons, long-interspersed nuclear element and long-terminal repeat retrotransposons. The genome of the club-legged grasshopper shows high degree of synteny with the locusts *Schistocerca gregaria* and *Locusta migratoria*, and the analysis strongly indicates 3 autosome–autosome centric fusions in Gomphocerinae. The genome offers a valuable resource for grasshopper genomics and for exploring the genetic basis of a transspecies color polymorphism.

## Introduction

Genomes of Orthoptera (grasshoppers, crickets, and bush-crickets) are interesting for at least 4 reasons. First, they represent the largest genomes of all insects (Hanrahan and Johnston 2011; Mao et al. 2020; Yuan et al. 2021; Sun et al. 2023) and are thus of interest to genome size evolution. Second, they have a dynamic karyotype, with several known cases of major chromosomal rearrangement (Palacios-Gimenez et al. 2025), including the formation of newly evolved neo-sex chromosome systems (Castillo et al. 2010) and a high abundance of supernumerary B chromosome (Palestis et al. 2004). They are thus of interest for studying genomic organization and stability. Third, Orthoptera feature one of the most phylogenetically widespread cases of a transspecies color polymorphism, with green and brown color morphs coexisting within populations in about 30% of the species (Schielzeth 2020). Genomic resources will facilitate the quest for the genomic basis of this shared polymorphism. Fourth, many species are short winged and thus sedentary but develop long wings and the ability to disperse under unfavorable conditions. They are thus model systems for the study of phenotypic plasticity and dispersal polymorphisms (Cabon et al. 2025).

Orthopteran genomes are known to be large, variable in size and rich in repetitive DNA sequences (Palacios-Gimenez et al. 2020; Shah et al. 2020; Hawlitschek et al. 2023; Sun et al. 2023; Liu et al. 2024). This has hampered the assembly of Orthopteran genomes using short-read sequencing technologies. The situation is currently changing with the availability of long-read sequencing technologies, yet fully phased genome assembly still represents a challenge for very large and repeat-enrich genomes like those of orthopterans. To date, 15 chromosome-level and 8 scaffold-level reference genome assemblies have been published for Orthoptera (https://www.ncbi.nlm.nih.gov/datasets/genome/?taxon=6993). Most of the assemblies come from crickets (families Gryllidae, Trigoniidae, Myrmecophilidae) with relatively small genomes (0.6–2.3 Gb). Furthermore, the genomes of 2 swarming locusts (*Locusta migratoria*, 6.3 Gb, and *Schistocerca gregaria*, 8.7 Gb) have been sequenced along with 5 other species of *Schistocerca* (8.5–9.1 Gb). Outside crickets and the 2 locust genera, only 6 reference genomes have been assembled (2 Ensifera, 6.4–9.0 Gb, 3 Tetrigidae 1.0–1.7 Gb, 4 lines of a species of Morabinae 3.75–4.1 Gb). This means that the available genomes do not cover the phylogenetic diversity of Orthoptera. In particular, no genome from the subfamily Gomphocerinae has been sequenced and assembled, even though Gomphocerinae represent one of the most species-rich clades with among the largest insect genomes (Cigliano et al. 2025).

**Fig. 1.**
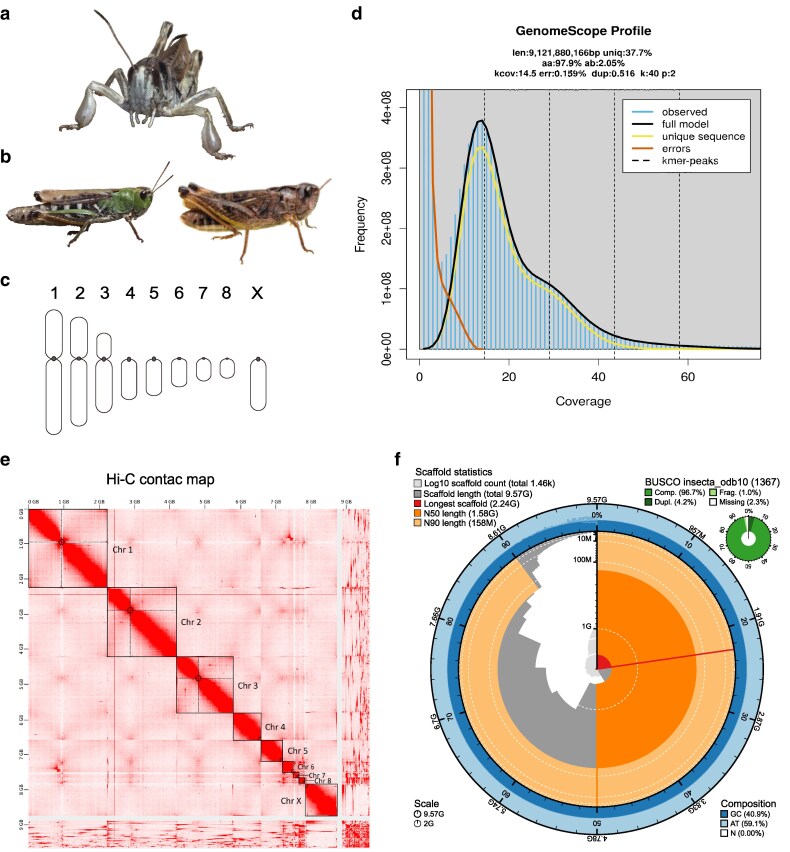
a) Male club-legged grasshopper *G. sibiricus* showing its unique swollen front-leg clubs. b) Female club-legged grasshoppers illustrating the green–brown polymorphism that is shared with many other species. c) Schematic drawing of the haploid male karyotype of club-legged grasshopper. Dots indicate positions of the centromere on each chromosome. Chromosomes are sorted by size and centromere position. The karyotype consists of 3 pairs of large metacentric autosomes (chr1 to chr3), 5 small-medium acrocentric autosomes (chr4 to chr8), and the acrocentric X chromosome. d) GenomeScope2 profile plot for k-mer 40 with associated summary statistics below the header. e) Hi-C contact map indicates 9 super scaffolds (indicated by black boxes), corresponding to 9 chromosomes of the club-legged grasshopper. Circles and dashed lines indicate the putative location of the centromeres. f) Summary and evaluation of the genome assembly, including genome assembly and scaffold statistics (depicted in a snail plot), along with a BUSCO (insecta_odb10) assessment displayed at the top right.

We present a reference chromosome-level annotated genome assembly for the club-legged grasshopper *Gomphocerus sibiricus* (Caelifera, Acrididae: Gomphocerinae, Fig. 1a-b). The species is green–brown polymorphic in both sexes and is unique among grasshoppers in featuring a marked sexual dimorphism in front-leg morphology (Valverde et al. 2018). Males of this species have a haploid chromosome set of n = 8A + X0 (Gosálvez and López-Fernández 1981) (Fig. 1c). Their genome includes 8 autosomes—3 large metacentric and 5 smaller acrocentric chromosomes—and follows an X0 sex-determination system. In this system, females are homozygous (XX), while males are hemizygous (X0) and lack a Y chromosome (Gosálvez and López-Fernández 1981). This haploid karyotype aligns with other species in the Gomphocerinae subfamily (Cabrero and Camacho 1986) but differs of the standard n = 11A + X0 found in most male Acrididae grasshoppers (John and Hewitt 1966; Palacios-Gimenez et al. 2025).

To provide a foundation for studying transspecies color polymorphism and genome evolution in species with large genomes, we generated a chromosome-level assembly of the club-legged grasshopper. Using PacBio HiFi long-reads and high-throughput chromatin conformation capture (Hi-C) for long-range scaffolding, we generated a 9.57 Gb assembly spanning 9 major scaffolds, covering 42,655 predicted protein-coding genes and 81.69% repetitive DNA sequences. The assembly is highly contiguous with a scaffold N50 of 1.58 Gb and a BUSCO completeness score of 96.7%. This high-quality genome provides an essential resource for understanding genomic architecture and evolutionary dynamics in grasshoppers.

## Methods

### Taxon sampling, DNA extraction, and sequencing

Males of the club-legged grasshopper (*G. sibiricus*) were collected in 2022 in north of the Col du Galibier (Savoie, France). Entire individuals were flash frozen in liquid nitrogen and preserved at −80 °C until DNA extraction. A total of 3 males were sequenced using different platforms: (i) we sequenced the genome of one male using PacBio HiFi long-read sequencing (12 Sequel SMRT cells 1 M on a PacBio Sequel II system) that produced 17,674,582 HiFi raw reads (283.1 Gb, genome coverage 28.6×). Mean HiFi read length was 16,013 bp; median HiFi read quality was 10; and mean HiFi number of passes was Q33; (ii) 2 male individuals were used to generate paired-end chromatin conformation capture (Hi-C) libraries using the Omni-C kit (Cantata Bio), following the manufacturer's protocol for nonmammalian samples (version 1). The Hi-C libraries were sequenced on an Illumina NovaSeq 6000 S4 flowcell, yielding a total of 2,257.7 million read pairs (2 × 150 bp). PacBio HiFi and Hi-C library preparation and sequencing were performed at the Uppsala Genome Center (UGC), Science for Life Laboratory (SciLifeLab), Department of Immunology, Genetics and Pathology, Uppsala University, Sweden.

### Genome size estimation

We used GeneScopeFK (https://github.com/thegenemyers/GENESCOPE.FK), a modified version of GenomeScope v2.0 (Ranallo-Benavidez et al. 2020), to estimate the genome size, genome coverage, and heterozygosity based on PacBio HiFi long-read sequencing with a k-mer size of 40.

### Genome assembly and Hi-C scaffolding

We assembled the PacBio HiFi long-read data into a primary assembly using Hifiasm v0.16.1 (Cheng et al. 2021) with default parameters and removed foreign contaminants with NCBI FCS-GX v0.5.4 (Astashyn et al. 2024). We then eliminated haplotigs and contig overlaps using Purge_Dups v1.2.6 (Guan et al. 2020). Hi-C reads were preprocessed and mapped to the decontaminated and purged primary assembly with Pairtools (Open2C et al. 2024), followed by scaffolding with YaHS v1.2.2 (Zhou et al. 2023). We generated a Hi-C contact map using JuicerTools v1.11.08 (Durand et al. 2016) and visualized the map with Juicebox v1.11.08 (Durand et al. 2016) to manually curate and correct the boundaries of the superscaffolds. We assessed the completeness of the chromosome-level assembly using BUSCO v5.5.0 (Manni et al. 2021) with the insecta_odb10 dataset (n = 1,367).

### X chromosome identification

In the male karyotype of the club-legged grasshopper, the X chromosome is present in a single copy, while each autosome is diploid. In this system, the X chromosome in male sequencing reads is thus expected to show half the coverage of the autosomes. We thus identified the X chromosome based on read coverage in PacBio HiFi long-read sequencing. We mapped the PacBio HiFi reads onto the newly generated chromosome-level assembly using minimap2 v2.26-r1175 (Li 2018) with default parameters. After mapping, we sorted the output BAM files by coordinate with SAMtools v1.20 (Danecek et al. 2021) and calculated the coverage in 1 Mb windows for each chromosomal scaffold with mosdepth v0.3.3 (Pedersen and Quinlan 2018) with the “–mapq 60' flag.

### Synteny with published grasshopper genomes

We used AnchorWave v1.2.5 (Song et al. 2022) to identify collinear regions between the genomes of the club-legged grasshopper and the desert locust *S. gregaria* (NCBI RefSeq assembly GCF_023897955.1), as well as between the club-legged grasshopper and the migratory locust *L. migratoria* (Li et al. 2024). AnchorWave performs whole-genome duplication–informed collinear anchor identification between genomes and performs base pair–resolved global alignment for collinear blocks using a 2-piece affine gap cost strategy. First, we took the desert locust genome sequence and gene annotation as input and extracted the full-length coding sequences to serve as anchors using the gff2seq function in AnchorWave. Second, we used minimap2 (Li 2018) in AnchroWave to lift over the start and end position of the desert locust full-length CDS to the club-legged grasshopper genome, with the settings -x splice -t 16 -k 12 -a -p 0.4 -N 20. Third, we used the proali function (settings -R2 -Q 1 -ns) in AnchorWave to identify collinear regions between the genomes. The outputs of this function are end-to-end sequence alignments for each collinear block that were used for plotting in R v4.3.0 (R Core Team 2022). Finally, we repeated all the aforementioned steps using the genomes of the club-legged grasshopper and the migratory locust *L. migratoria* in place of *S. gregaria*.

### Repeat annotation

We used Earl Grey v5.1.0 (Baril et al. 2024) to predict repetitive DNA sequences *de novo* in the new chromosome-level assembly and retrieve a nonredundant species-specific repeat library. We used the repeatlib_filtering_workflow to filter the repeat library for non-TEs resembling proteins (source code: https://github.com/NBISweden/repeatlib_filtering_workflow) and parsed the filtered repeat library through TEtrimmer (Qian et al. 2025) to assist in the manual curation of the repeat library. The curated library is available on Figshare (https://doi.org/10.6084/m9.figshare.29148746) and is currently being submitted to the Dfam database (Storer et al. 2021). After curation, we merged the curated nonredundant species-specific repeat library with Arthropoda consensus sequences from Repbase (Bao et al. 2015). Finally, we used this repeat library to annotate the chromosome-level assembly with RepeatMasker v4.1.0 (Smit et al. 2019). We used the script *calcDivergenceFromAlign.pl* from RepeatMasker utils to calculate the divergence between repeats and their consensus sequences using the Kimura 2-parameter distance while accounting for the presence of CpG sites.

### Genome annotation

The repeat-masked genome was used for gene model annotation with BRAKER3 v3.0.8 (Gabriel et al. 2024). To support gene prediction, we used the paired-end RNA-seq reads previously generated for the club-legged grasshopper deposited in NCBI under the BioProject PRJNA525981 and PRJNA1241690 together with Arthropoda protein data from OrthoDB v11 (Kuznetsov et al. 2023). The published RNA-seq samples (Shah et al. 2019) in the BioProject PRJNA525981 included the pool of 5 individuals: 1 imago brown female, 1 imago green female, 1 imago brown male, 1 imago green male, and 1 last-instar green female (accession number SRX5491242 and SRX5491243). RNA-seq samples in the BioProject PRJNA1241690 included 11 adult females (accession number SAMN47561012-SAMN47561022) and 11 adult males (accession number SAMN47561023- SAMN47561033), with RNA extracted specifically from the thorax tissue.

We aligned the each published paired-end RNA-seq dataset to the repeat-masked genome using HISAT2 v2.2 (Kim et al. 2015) with the “–dta” parameter under default settings. We then sorted the resulting BAM files with SAMtools v1.14 and used them alongside the Arthropoda protein database to run BRAKER3. To complement BRAKE3's predictions, we used Helixer v0.3.5 (Holst et al. 2023 Feb 6) with the flags “–lineage invertebrate –subsequence-length 213,840 –overlap-offset 106,920 –overlap-core-length 160,380 –peak-threshold 0.9 –batch-size 16.” Finally, we combined the annotation files from BRAKE3 and Helixer using the *agat_sp_complement_annotations.pl* script from the AGAT v1.4.0 toolkit (Dainat et al. 2024).

## Results and discussion

### Genome size and genome assembly

Using GenomeScope2 with a k-mer length of 40, we predicted genome size of the club-legged grasshopper to be 9.12 Gb with 2% heterozygosity (Fig. 1d). This estimate is slightly lower than the 10.15 Gb genome size estimated from the testis using Feulgen densitometry (Gosalvez et al. 1980) and the 10.43 Gb estimated from brain cells using flow cytometry (Shah et al. 2020).

The decontaminated and purged primary PacBio HiFi assembly consisted of 3,752 contigs (N50 contig 6.52 Mb) with a total assembly size of 9.57 Gb (Table 1). Scaffolding with Hi-C data resulted in final assembly containing 9 chromosome models (N50 scaffold 1.58 Gb, Fig. 1e; Table 1). The assembly size slightly exceeds the estimates from the k-mer analysis but is lower than the estimates from Feulgen densitometry and flow cytometry. This chromosome-level assembly represents the largest published chromosome-level insect genome to date. The 9 chromosomal models match the species karyotype (Gosálvez and López-Fernández 1981) and comprise scaffolds of 158 to 2,237 Mb in size, covering 93% of the whole assembly (Fig. 1e; Table 1); unplaced scaffolds consisted of 7% of the total assembly size. BUSCO analysis of the chromosome-level assembly revealed C:96.7% [S:92.5%, D:4.2%], F:1%, M:2.3%, n:1,367 (Fig. 1f; Table 1), indicating the assembly well–captured protein-coding genes.

**Table 1.** Summary statistics for the final assembly, gene, and repeat annotation

Assembly statistics	
Assembly size (Gb)	9.57
Scaffolds	1,462
N50 scaffold (Gb)	1.58
L50 scaffold count	3
Contigs	3,752
N50 contig (Mb)	6.52
Assembly in scaffolded contigs (%)	92.7
Assembly in unscaffolded contigs (%)	7.3
BUSCO	C:96.7% [S:92.5%, D:4.2%], F:1%, M:2.3%, n:1367
**Annotation statistics**	
Protein-coding genes	43,655
mRNA	43,885
Single exon gene	9,799
Mean exons per mRNA (bp)	5.8
Mean exon length (bp)	292
Mean intron length (bp)	18,564
BUSCO	C:93.9% [S:73.7%, D:20.2%], F:2.3%, M:3.8%, n:1367
**Repeat statistics**	
TE content (%)	71.63
* SINE (%)*	0.57
* LINE (%)*	21.01
* LTR (%)*	22.05
* DNA (%)*	22.57
* RC (%)*	7.95
* Unknown (%)*	5.18
Satellite DNA (%)	1.41
Simple repeats (%)	0.64
Low complexity	0.04
Total repeat content (%)	81.69

Comparing the metrics of the chromosome-level assembly of the club-legged grasshopper to the available *Schistocerca* grasshopper assemblies (range 8.5 to 9.1 Gb, scaffold N50 ranges 791.2 to 992.9 Mb) on NCBI (https://www.ncbi.nlm.nih.gov/datasets/genome/?taxon=7008) reveals that the club-legged grasshopper has a slightly larger genome size and higher contiguity compared with other *Schistocerca* genomes. The genome also stands out from all other so far published orthopteran genome assemblies in size and contiguity.

### X chromosome identification

**Fig. 2.**
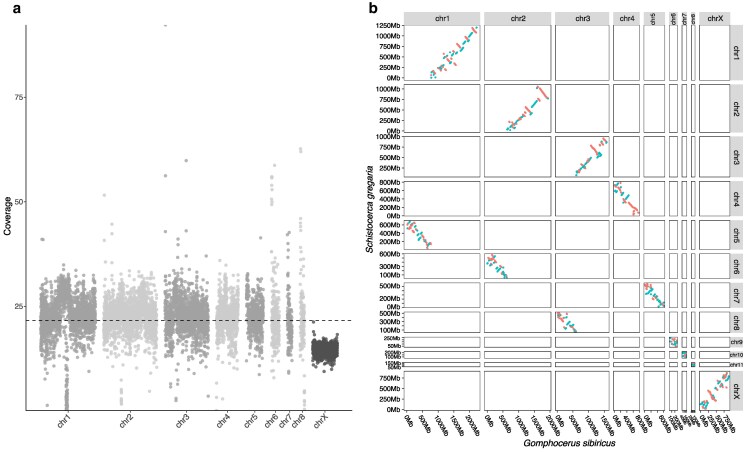
a) Genome-wide PacBio HiFi read coverage across 1 Mb windows is plotted along chromosomal positions in the club-legged grasshopper *G. sibiricus* genome. The dashed black line indicates the average read coverage across the autosomes. The reduced coverage on one of the scaffolds identifies the scaffold as the sex-linked X chromosome. b) Synteny of Acridid genomes inferred from collinear anchors between the desert locust *S. gregaria* and the club-legged grasshopper chromosome-level assemblies. Each dot is plotted based on the start coordinate of the reference genome (*S. gregaria*) and query genome (*G. sibiricus*) of each anchor. Collinear anchors on the same strand are shown in blue; anchors on the opposite strands are shown in red.

We identified the X chromosome by analyzing genome coverage from PacBio HiFi long-read sequencing. Most chromosome models maintained consistent coverage across 1 Mb windows, but the fourth-largest chromosome (size 899 Mb) shows reduced coverage compared with the remaining chromosome models (Fig. 2a). This chromosome model thus represents the assembly of the X chromosome. We assigned the remaining 8 chromosomes as chr1 to chr8 in a descending order of size, as shown earlier (Fig. 1c, Fig. 1e).

### Synteny of chromosomes with other Orthoptera

Male club-legged grasshoppers have a haploid karyotype of n = 8A + X0, which includes 3 large metacentric chromosomes, characterized by a centromere located near the midpoint of the chromosome, resulting in a short and a long arm. The conformance also applies to all European Gomphocerinae grasshoppers characterized so far (Cabrero and Camacho 1986; Palacios-Gimenez et al. 2025). In contrast, most other subfamilies of Acrididae grasshoppers have a male haploid karyotype of n = 11A + X0, where all chromosomes are acrocentric, featuring a single arm with centromeres located near the periphery (Palacios-Gimenez et al. 2025).

Three autosome–autosome centric fusions (A-A centric fusions) putatively reduced the chromosome number in gomphocerine grasshoppers (including the club-legged grasshopper), a trait shared by half of the analyzed gomphocerine grasshopper species (including all European species), suggesting it originated in a common ancestor (Cabrero and Camacho 1986). Using AnchorWave, we identified 14,607 collinear anchors between the desert locust *S. gregaria* (Acrididae: Cyrtacanthacridinae) and our novel assembly. The same set of anchors was also found between the migratory locust *L. migratoria* (Acrididae: Oedipodinae) and our assembly. Our analysis showed that A-A centric fusions between chr1-chr5, chr2-chr6, and chr3-chr8 chromosomes (ordered by the size in the locusts) gave rise to the 3 largest autosomal metacentric chromosomes in the club-legged grasshopper (Fig. 2b, Supplementary Fig. 1). These findings confirm the role of A-A centric fusions in driving karyotype diversity across grasshopper genomes.

Furthermore, we found that the X chromosome of the club-legged grasshopper is homologous to that of the locusts (Fig. 2b, Supplementary Fig. 1), confirming its shared ancestry with the X chromosome of other insects (Toups and Vicoso 2023; Li et al. 2024). We found many rearrangements, mostly inversions, within chromosomes, but no evidence of any cross-chromosome translocations (Fig. 2b, Supplementary Fig. 1). This seems remarkable given the divergence time of 45 My (Song et al. 2020).

### Centromere identification

The Hi-C map showed increased contacts between 1 region each in the center of the 3 large chromosome models and at 1 end of the 6 others chromosome models. These contacts are indicative for the approximate location of the centromeres (Fig. 1e). For the 3 metacentric chromosomes, the putative centromere is located slightly off-center, consistent with the karyotype description. In contrast, for the 5 smaller acrocentric autosomes, the contact map suggests the centromere is positioned at the distal end and for the acrocentric chromosome X at the proximate end (Fig. 1e).

### Repeat annotation

We *de novo* identified repetitive DNA sequences and constructed a curated nonredundant species-specific repeat library containing 4,408 consensus sequences. The proliferation of repetitive DNA sequences drives the large genome size of the club-legged grasshopper (Shah et al. 2020), a pattern that correlates with genome size variation across the Tree of Life (Gregory 2005; Hua-Van et al. 2011; Cabral-de-Mello and Palacios-Gimenez 2025). Repetitive DNA accounts for 81.69% of the club-legged grasshopper genome assembly, including 22.57% DNA transposons, 22.05% long-terminal repeat (LTR) retrotransposons, 21.01% long-interspersed nuclear elements (LINE), 7.95% rolling-circles (RC), 0.57% short-interspersed nuclear elements (SINE), 0.25% Penelope-like elements (PLE), 1.41% satellite DNA, and only 5.18% of unknown repeats (Table 1). The repetitive DNA content of the club-legged grasshopper (81.69%) is comparable to those of other previously assembled grasshopper genomes, which range from 60% to 80% (Palacios-Gimenez et al. 2020; Verlinden et al. 2020; Jayaprasad et al. 2024; Li et al. 2024; Li et al. 2024). The fraction of satellite DNA and RC is substantially reduced as compared to previous estimates for the club-legged grasshopper (Shah et al. 2020), likely due to long-read sequencing combined with improved curation and annotation. After subtracting repetitive DNA sequences, the club-legged grasshopper's nonrepetitive genome spans 1.75 Gb, making it remarkably larger than that of most other insects (Cong et al. 2022; Cabral-de-Mello and Palacios-Gimenez 2025).

**Fig. 3.**
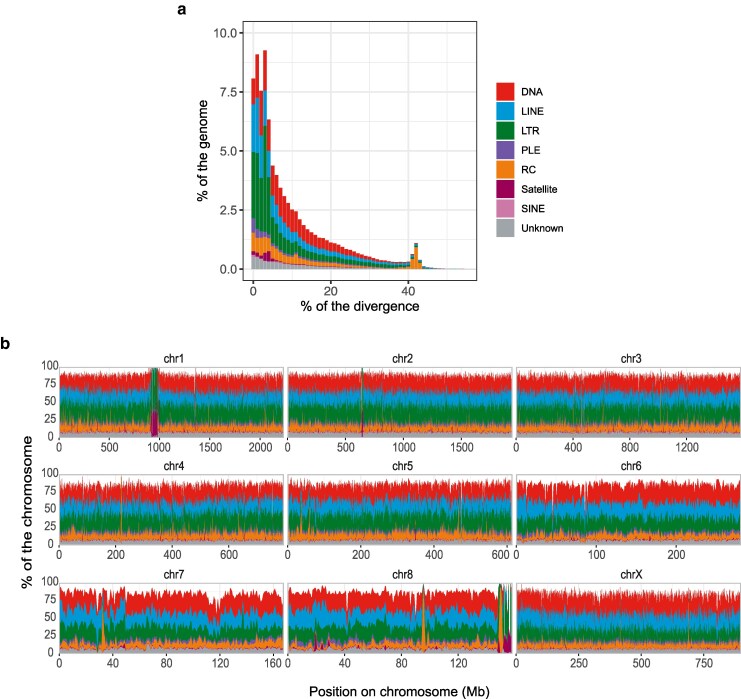
Assembly-based repeat landscape analysis in the club-legged grasshopper *G. sibiricus* genome. a) The divergence between repetitive DNA copies and their consensus sequences is shown on the X-axis as genetic distance calculated using the Kimura 2-parameter distance. The percentage of the genome assembly occupied by repetitive DNA sequences in each class is shown on the Y-axis. b) The percentage of repeat-derived base pairs shown per window of 1 Mb along each assembled chromosome, following the same color scheme as in (a).

Estimates of sequences divergence within repeat classes indicates 2 periods of repetitive DNA proliferation. A recent repetitive DNA burst occurred within the 0% to 10% divergence range, involving all major repetitive DNA classes (Fig. 3a). Another burst, primarily consisting of RC elements, appeared within the 41% to 43% divergence range, suggesting these represent older and degenerated RC copies (Fig. 3a).

### Gene annotation

We predicted the structure of 43,655 putative protein-coding genes and 43,885 mRNA in the club-legged grasshopper chromosome-level assembly (19,410 genes identified by BRAKER3 and 30,992 genes identified by Helixer), with an average exon size and intron size of 292 (median 143 bp) and 18,564 bp (median 6,551 bp), respectively (Table 1). BUSCO score on the predicted transcripts are 94% (Table 1), indicating the genome captures almost the entire gene space. Overall, 6.4% of the predicted protein-coding genes are found in the unplaced scaffolds (only 2% of the BRAKER predicted genes). The total of 43,655 predicted protein-coding genes in club-legged grasshopper is substantially more than those reported in the migratory locust *L. migratoria* (26,636) (Li et al. 2024) and the desert locust *S. gregaria* (18,815) (Verlinden et al. 2020). This probably resulted from the use of multiple annotation platforms to enhance gene prediction accuracy. The average intron size in the club-legged grasshopper genome is substantially larger than the average intron size of ∼1,000 bp in *Drosophila melanogaster* (Hoskins et al. 2015) and even larger than the average intron size of ∼7,000 bp (median ∼1,700 bp) in humans (Piovesan et al. 2019). Intron gigantism therefore contributes to the large genome size of the club-legged grasshopper.

## Data Availability

The final assembly and raw data have been deposited at NCBI under the project accession number PRJNA1250961. The genome assembly is available under accession number JBPBLO000000000. PacBio HiFi long-read sequencing data can be found under accession numbers SRX29045381, SRX29045382, SRX29045386-SRX29045393, SRX29073929, and SRX29073928. Hi-C sequencing data are available under accession numbers SRX29045383-SRX29045385. The associated files for this manuscript are available on the figshare database: Supplementary Fig. 1 (https://doi.org/10.6084/m9.figshare.29476994), Gene annotation models (https://doi.org/10.6084/m9.figshare.29148779) RepeatMasker annotation (https://doi.org/10.6084/m9.figshare.29148836), curated nonredundant species-specific repeat library (https://doi.org/10.6084/m9.figshare.29148746). Supplemental material available at G3 online.
